# Anthraquinone Content in Noni (*Morinda citrifolia* L.)

**DOI:** 10.1155/2013/208378

**Published:** 2013-08-26

**Authors:** Rainer W. Bussmann, Lothar Hennig, Athanassios Giannis, Jutta Ortwein, Toni M. Kutchan, Xi Feng

**Affiliations:** ^1^William L. Brown Center, Missouri Botanical Garden, P.O. Box 299, St. Louis, MO 63166-0299, USA; ^2^Institute of Organic Chemistry, University of Leipzig, Johannisallee 29, 4103 Leipzig, Germany; ^3^Institute of Pharmacy, University of Leipzig, Brüderstraße 34, 4103 Leipzig, Germany; ^4^Donald Danforth Plant Science Center, 975 N. Warson Road, St. Louis, MO 63132314-587-1473, USA

## Abstract

Noni has been used in traditional medicine and as food for thousands of years. While the fruits serve as food and internal medicine, leaves were traditionally used only topically. In recent years, concern regarding the possible content of anthraquinones in noni has led to scrutiny by the European Food Safety Authority. Little research existed on the content of anthraquinones in different noni preparations, with no information about the potential effect of harvest and preparation methods. Our research focused on lucidin, alizarin, and rubiadin, the most important anthraquinones from a health perspective. We found that the production process (fermentation/juice production versus drying/lyophilization) has no effect on the anthraquinone content. The source product, however, does have implications: noni fruit puree from which seeds had been removed as well as consumer products produced from such puree had no detectable amounts of any anthraquinones. Products that did contain seed or leaf material in all cases did contain partly significant amounts of anthraquinones. To alleviate safety concerns, we suggest that noni products, whether fermented or unfermented juice or powder, should be derived only from fully ripe noni fruits, and that any seed material needs to be removed during the production process.

## 1. Introduction

Noni (*Morinda citrifolia* L., Rubiaceae) probably originated in the Indonesian archipelago and was widely distributed during the Polynesian migration as one of the important “canoe plants,” finally reaching French Polynesia and Hawai'i. Traditionally, the fruits were used as food a treatment for and intestinal problems, while the leaves served for the treatment of wound infections, arthritis, swellings, and similar conditions [1, 2]. Recent research indicated anti-inflammatory and antioxidant properties [3, 4]. During the last decade, noni, mostly marketed as a fermented juice, has become a widely traded food supplement worldwide, based on health claims related to some of its compounds, in particular flavonoids [5–7].

Starting in 2005, some reports on the hepatotoxicity of noni preparations raised health concerns [8, 9] and led the European Food Safety Authority to conduct further research. A conclusion of this work was that the regular intake of noni juice would most likely not cause any toxic effects [10]. Analyses sponsored by Tahitian Noni, the main global provider of noni juice, reported no toxicity from consumption of the product [11–13]. The main health concerns were based on the possible content of carcinogenic anthraquinones, in particular alizarin, rubiadin, and lucidin in noni. Anthraquinone and its derivatives are common aromatic compounds in plant pigments and are used to make dyes and paper, as well as bird repellants. The US National Toxicology Program investigations concluded that anthraquinones caused cancer of the kidney, urinary bladder, liver, and thyroid in rats and mice [14]. Comparative studies reported the presence of these compounds in madder roots (*Rubia tinctorum*, Rubiaceae) and animal models led to the conclusion that these compounds could possibly have genotoxic and carcinogenic effects [15, 16]. The same compounds were reported from the wood [17, 18], stems [19], and roots [7, 20–22] of *M. citrifolia.* Smaller amounts were reported from flowers [23], leaves [24], and to some extent from fruits [23, 25–27]. However, no studies had attempted to quantify anthraquinones in noni preparations until Deng et al. [6] developed a method to elucidate the anthraquinone content of noni based on noni pulp samples from Tahitian Noni and some noni leaf tea products. None of the tested materials contained anthraquinones in higher amounts. However, these studies did not provide any indication as to under which production conditions plant material in commerce might in fact contain higher anthraquinone amounts, and if the removal of certain plant compounds from preparations might lower the possible anthraquinone content.

The present study attempted to remedy this situation by examining the real content of anthraquinones in different noni preparations and to include information about the potential effect of harvest and preparation methods. Our research focused on alizarin, lucidin, and rubiadin, the most important anthraquinones from a health perspective, and used a variety of different preparations (fermented and unfermented; juice and powders; with and without seeds, leaves, and leaf fragments).

## 2. Materials and Methods

### 2.1. Materials for Sample Preparation

For high-performance liquid chromatography (HPLC) analysis and liquid chromatography-mass spectrometry (LC-MS), fresh plant material was wild collected in Honolulu (O'ahu) and Kalapana (Hawai'i) and identified by researchers at the William L. Brown Center at the Missouri Botanical Garden. Samples for commercially sold noni preparations were supplied by Arogya Inc., Honolulu. Lucidin and rubiadin (pro analysi—analysis grade)**  **were obtained from Cfm Oskar Tropitzsch e.K., Marktredwitz, Germany. Reference chromatograms and ultraviolet (UV) spectra for lucidin, rubiadin, and anthraquinone were established.

### 2.2. Sample Preparation

#### 2.2.1. HPLC Analysis****

*Plant Material*. Seedless Arogya Noni (noni fruit pulp, freeze dried, with no seeds, and no skin parts), 2 samples of 0.5 g. Subsequently addition of 20 ml ethylacetate (HPLC grade), 30 min. agitation, and filtration of solids (filter paper soaked ethylacetate). Reduction to dryness in SPE-chamber and reconstitution of product in 500 ml methanol, filtration (45 min), folowed by induction in HPLC [6]. HPLC was conducted with a Macherey-Nagel, Nucleodur C18 Pyramid, 5 mcl column, with 0.1% trifluoroacetic acid (p.a. HPLC gradient grade) as solvent A and acetonitrile (p.a. HPLC gradient grade) as solvent B at 25°C. Flux velocity was set at 1.0 mL/min, detection set at 275 nm and 410 nm.

#### 2.2.2. Liquid Chromatography-Mass Spectrometry (LC-MS)

Acetonitrile and formic acid (HPLC grade, Acros Organics), ammonium formate and tetrahydrofuran (HPLC grade, Sigma-Aldrich). Alizarin (dye content 97%) and purpurin (dye content 90%) were purchased from Sigma-Aldrich, and lucidin and rubiadin were obtained from the laboratory of natural product collection at the Donald Danforth Plant Science Center.

Dried and powdered samples (1.25 g) were stirred in 25 mL of ultrapure water for 1 h at 45°C. After cooling to room temperature, 50 mL of tetrahydrofuran-water-formic acid (1 : 1 : 0.005) was added and the mixture was stirred for an additional 30 min at room temperature. The supernatant was collected and filtered through a 0.2 mcm, 25 mm diameter PVDF membrane filter (PALL Life Sciences). Fresh serum samples (5 g) were freeze-dried with a lyophilizer, and then the same sample preparation procedure as for dried and powdered samples was applied. Fresh fruit samples (2.5 g) were freeze-dried with a lyophilizer, and ground into powder with a mortar and pestle. The same sample preparation procedure as for dried and powdered samples was then applied.

Samples were initially analyzed by HPLC at 254 nm and 280 nm. An LC-MS method (MRM-Multiple Reaction Monitoring) was developed to detect and quantify the anthraquinones in all samples. The liquid chromatography-mass spectrometry (LC-MS) system consisted of a CTC Pal autosampler (LEAP Technologies), a Shimadzu LC-20AD liquid chromatograph, and a 4000 QTRAP mass spectrometer (Applied Biosystem). Separation of (30 mcl) samples was achieved on a LiChroCART 250-4 HPLC column (Merck, 5 *μ*m, LiChroaphor 60 RP select B) combined with a LiChroCART 4-4 HPLC cartridge (Merck, 5 *μ*m, LiChroaphor 60 RP select B). The mobile phase total flow rate was set to 1.0 mL/min with binary gradient elution, using an ammonium formate formic acid buffer (0.2 M, pH 3) as solvent A and 90% acetonitrile as solvent B.

Compound-dependent parameters are described in Table 1. The Turbo V Ion Source (TIS) was used in negative mode and the following source parameters were used: CUR 30, CAD high, IS-4500, TEM 500, GS1 50, GS2 55, and EP-10 (Table 1).

## 3. Results

### 3.1. HPLC

**Figure 1:**
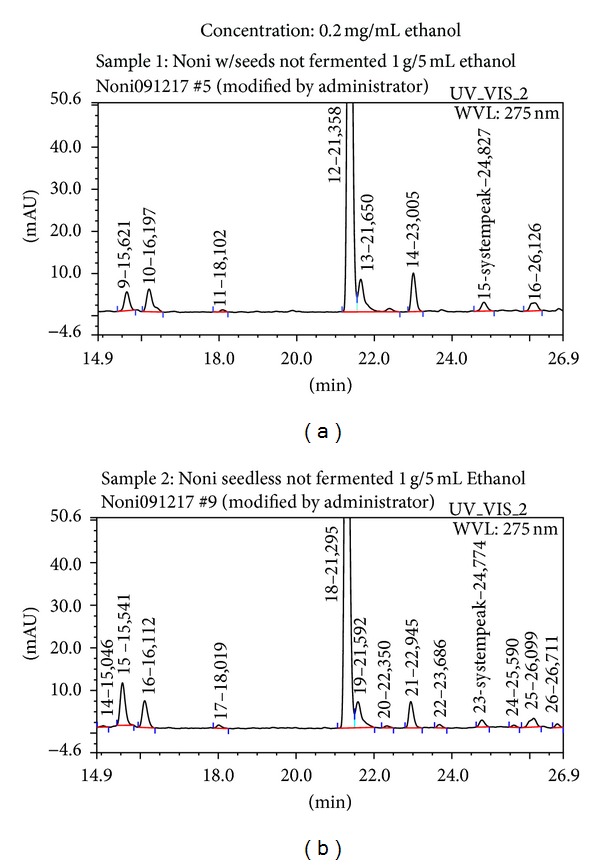
HPLC of representative noni samples. None of the samples contained detectable amounts of rubiacin or lucidin.

None of the samples contained detectable amounts of anthraquinone, lucidin, or rubiadin (Figure 1).

Additionally, HLPC studies confirmed that lucidin is not a very stable compound. It was already observed, in 1984, that the formation of lucidin *ω* (*ω* = Greek  Omega) ethers could be artifacts derived from lucidin as methanol and chloroform were used as solvents for extraction, although there was no direct proof for this possibility [28]. Otherwise, authors [29] have shown in 2010 that lucidin and its derivatives can be activated in the metabolic pathway to react with the DNA [28].

Storing HPLC solution of lucidin in acetonitrile which was treated with a small amount of ethanol for better solubility, the formation of a new product was observed within several days. A retention time very similar to rubiadin was established for this compound in the chromatogram. Obviously this product is identical to the compound described as unknown anthraquinone [13]. Careful workup, isolation, and structural investigation by ^1^H-NMR and high resolution MS (negative mode) showed that this compound is ibericin (lucidin ethyl ether).

### 3.2. LC-MS/MS

The results presented in Table 2 indicate that all noni samples containing fragments of leaves or fruit skin did show traces of various anthraquinones. In clear contrast, samples that were produced under the removal of fruit skin and free of leaf material did not contain detectable amounts of anthraquinones. The inclusion of seed material did not influence the anthraquinone content.

## 4. Discussion

The concern regarding the possible content of anthraquinones in noni products has led to scrutiny by the European Food Safety Authority. The present study indicates that the production process (fermentation and juice production versus drying or lyophilization) has no effect on the anthraquinone content.

Fruit ripeness as such also did not have any influence on anthraquinone content. However, it is to be noted that the removal of seeds and fruit skin from fully ripe fruits is much easier than from unripe noni. This has serious implications on the production process.

The source product, however, does have implications: noni fruit puree from which seeds and skin fragments had been removed as well as products (juice as well as capsules) produced from such puree had no detectable amounts of any anthraquinones. In contrast, products that did contain fruit skin or leaf material did contain partly significant amounts of all anthraquinones in all cases.

To alleviate potential safety concerns, we suggest that commercial noni products in the market, whether fermented or unfermented juice or powder, should be derived only from fully ripe noni fruits, and that any seed material needs to be removed during the production process.

## Figures and Tables

**Table 1:** Compound-dependent parameters for the LC-MS/MS method.

Analyte	Collision energy (V)	Declustering potential (V)	Collision cell exit potential (V)	MRM transition	MRM transition
Alizarin	−40	−80	−10	239/211	239/167
Rubiadin	−35	−100	−10	253/225	253/209
Purpurin	−40	−90	−10	255/227	/
Lucidin	−30	−75	−10	269/251	/

**Table 2:** Anthraquinone content in Noni samples.

Sample ID	Lucidin 269/251	Total alizarin mg/kg	Purpurin 255/227	Rubiadin 253/225	
Sample_#1	/	/	/	/	Fresh noni seedless pulp, no skin
Sample_#2	/	/	/	/	Fresh noni seeded pulp, no skin
Sample_#3	/	0.152	/	Detectable	Fermented noni fruits
Sample_#4	/	/	/	/	Noni powder, seedless, and no skin
Sample_#5	/	0.279	/	Detectable	Ripe noni dried
Sample_#6	Detectable	0.337	/	/	Overripe noni dried
Sample_#7	/	0.781	/	/	Noni powder
Sample_#8	Detectable	0.334	/	/	Noni powder (unfermented)
Sample_#9	Detectable	4.655	/	Detectable	Noni powder (fermented)
Sample_#10	Detectable	0.365	/	Detectable	Noni powder
Sample_#11	Detectable	7.797	/	Detectable	Noni powder (fermented)
Sample_#12	Detectable	0.774	/	/	Noni powder
Sample_#13	Detectable	/	/	/	Noni powder
Sample_#14	/	8.612	/	Detectable	Noni powder (Peru)
Sample_#15	Detectable	0.725	/	Detectable	Noni powder
Sample_#16	Detectable	0.677	/	Detectable	Noni powder
Sample_#17	/	/	/	/	Juice, seedless, and no skin
Sample_#18	/	/	/	/	Juice, seedless, and no skin
Sample_#19	/	0.053	/	Detectable	Noni juice
Sample_#20	/	/	/	/	Noni juice, seedless, and no skin
Sample_#21	/	0.281	/	Detectable	Noni leaf tincture
Sample_#22	/	/	/	/	Noni tonic
Sample_#23	/	/	/	/	Maca/Cordia powder (comparison)
Sample_#24	/	/	/	/	Chilchos coffee (for comparison)
